# The Quintessence of Traditional Chinese Medicine: Syndrome and Its Distribution among Advanced Cancer Patients with Constipation

**DOI:** 10.1155/2012/739642

**Published:** 2012-06-24

**Authors:** Chung-Wah Cheng, Annie O. L. Kwok, Zhao-Xiang Bian, Doris M. W. Tse

**Affiliations:** ^1^Yan Chai Hospital, Hong Kong Baptist University Clinical Centre for Training and Research in Chinese Medicine (West Kowloon), Kowloon, Hong Kong; ^2^Department of Medicine & Geriatrics, Caritas Medical Centre, Kowloon, Hong Kong; ^3^Clinical Division, School of Chinese Medicine, Hong Kong Baptist University, Kowloon Tong, Hong Kong; ^4^Department of Medicine & Geriatrics, Intensive Care Unit (ICU), Caritas Medical Centre, Kowloon, Hong Kong

## Abstract

Constipation is a common problem in advanced cancer patients; however, specific clinical guidelines on traditional Chinese medicine (TCM) syndrome (*Zhang*) are not yet available. In this cross-sectional study, the TCM syndromes distribution and their common symptoms and signs among 225 constipated advanced cancer patients were determined. Results showed that 127 patients (56.4%) and 7 patients (3.1%) were in deficient and excessive patterns, respectively, while 91 patients (40.4%) were in deficiency-excess complex. The distributions of the five syndromes were: *Qi* deficiency (93.3%), *Qi* stagnation (40.0%), blood (*Yin*) deficiency (28.9%), Yang deficiency (22.2%), and excess heat (5.8%). Furthermore, age, functional status, and level of blood haemoglobin were factors related to the type of TCM syndrome. A TCM prescription with the functions on replenishing the Deficiency, redirecting the flow of *Qi* stagnation and moistening the dryness caused by the blood (*Yin*) deficiency can be made for the treatment of advance cancer patients with constipation. Robust trials are urgently needed for further justifying its efficacy and safety in evidence-based approaches.

## 1. Introduction

Traditional Chinese medicine (TCM) has been in use for curing diseases and promoting the health of human for thousands of years in China. Its theory is derived from Chinese ancient philosophy and completed as the result of long-term clinical practices. Nowadays, TCM becomes the main component of complementary and alternative medicine (CAM) and gains increasing attention and popularity in the world [1]. Syndrome (*Zheng*), also called pattern, is the key concept in TCM theory [2]. It is a summary of the cause, nature, and location of the pathological change at a certain stage of disease [3] and is the foundation for making diagnosis and prescription. Chinese Medicine practitioners make the differentiation on the basis of all symptoms and signs collected by the four classic diagnostic methods, that is, observation, inquiry, smelling/listening, and palpation. Patients with same disease can present in different syndromes. Oppositely, patients with different diseases can present in same syndrome [4]. Due to the complexity and diversity of syndrome, TCM experts have attempted to quantize the description and standardize the terminology of syndrome since 1980s [5, 6]. Certain practice guidelines for difference syndromes of a specific disease are established as a reference for making diagnosis and prescribing treatment.

Constipation is a common problem in advanced cancer patients, which affects an estimated 32% to 87% of patients [7–10] and is only superseded by pain and anorexia [7]. Constipation is also a distressing symptom, and if untreated, can give rise to nausea, vomiting, abdominal distension, urinary retention, anal fissures, and even bowel obstruction [11]. However, the “best” treatment for constipation on both efficacy and safety remains an unresolved issue [12, 13]. According to the TCM theory, constipation can be broadly divided into excessive and deficient patterns based on the underlying aetiology. The excessive pattern is characterized by excess heat or pathological accumulation of *Qi *(stagnation), while the deficient pattern is characterized by the dryness from insufficient fluid lubrication in the form of blood (*Yin*) or lack of propulsion power from the deficiency of *Qi* or *Yang *[4, 14, 15]. Five common TCM syndromes can be summarized from the TCM references of Internal Medicine of Chinese Medicine [4], Criteria of Diagnosis and Therapeutic Effect of Diseases and Syndromes in Traditional Chinese Medicine [5], and Clinical Handbook of Internal Medicine [14, 15]. They are the intestine with excess heat, intestinal *Qi* stagnation, spleen deficiency with weakness of *Qi*, spleen-kidney *Yang* deficiency, and *Yin* deficiency with intestinal dryness. Each of them has its unique treatment principle and prescription. However, none of these references is specified on advanced cancer patients. The constitutions of these patients are different from general patients only with constipation; therefore, an epidemiological study on syndrome distribution is important for the establishment of practice guidelines in palliative cancer care.

In this present study, the TCM syndromes distribution and their common symptoms and signs among constipated advanced cancer patients are first determined. Besides, the impacts of patient demographics and opioids prescribed on TCM syndromes are also investigated. The results are important for tailor-made Chinese herbal formulation for the management of constipation for advanced cancer patients, and launching large-scale clinical study in future. In parallel, the bowel habits and the use of relieving measures are examined and reported in a separated article [16].

## 2. Materials and Methods

This study was a cross-sectional survey carried out in the palliative care units of Caritas Medical Centre and Our Lady of Maryknoll Hospital, which ran a comprehensive range of specialist palliative care services including inpatient, outpatient, home care, and day care services. Participants were interviewed by a registered Chinese medicine practitioner who possessed a degree in Chinese Medicine and with at least three-year working experience on clinical practice and TCM-related scientific researches. The whole study was conducted in accordance with the Declaration of Helsinki. Ethics approval was obtained from the Kowloon West Cluster Research Ethics Committee, Hong Kong Hospital Authority, and the study protocol was registered at ClinicalTrials.gov (NCT01399294).

### 2.1. Patients

All advanced cancer patients (aged 18 or above) under the care of palliative care unit of Caritas Medical Centre and Our Lady of Maryknoll Hospital who had constipation during the period from May 1, 2010 to July 31, 2010 were invited to participate in the study. Patients were recruited only from those who reported (1) on medications (laxatives, enemas, Chinese herbal medicine (CHM), and/or health supplements) for facilitating bowel movement; (2) constipation as two points or more by the constipation visual analogue scale (CVAS) (0: none to 7: most severe) [17]. Patients who were unable to communicate, cognitive impaired, put on colostomy bag, clinically diagnosed gastrointestinal obstruction, or at end-of-life (EOL) were excluded. Written or verbal informed consents were obtained before starting the interview, and all subjects were free to withdraw at any time from the study.

### 2.2. Questionnaire

The questionnaire was designed by the research team with Traditional Chinese Medicine professionals and Palliative Medicine specialists, comprising of three parts written in Chinese (See Appendix 1 available online at doi:10.1155/2012/739642). The first part consisted of patient demographics. Patient's age, gender, primary cancer, functional status as measured by Palliative Performance Scale (PPS), biochemical parameters from blood tests, and prescription of opioid were recorded by the Palliative Medicine specialists. The second part was about patients' perception of bowel function. The bowel habit, such as frequency, stool type, rectal measure, and laxatives/enemas required, was enquired. Besides, the severity of constipation was evaluated by the constipation visual analogue scale (CVAS). It was an 8-point ordinal rating scale, where 0-1 indicated no constipation, 2–4 indicated constipation, and 5–7 indicated severe constipation [18]. Specific analysis and interpretations about the correlation between the patient demographics and their bowel habits were reported with details in a separate paper [16].

The third part consisted of the TCM syndrome patterns as diagnosed by a registered Chinese Medicine practitioner. Five syndromes, that is, excess heat, *Qi* stagnation, *Qi* deficiency, *Yang* deficiency, and *Yin* deficiency, were simplified from the TCM references [4, 5, 14, 15]. The former two were in excessive pattern, while the latter three were in deficient pattern. Typical symptoms and signs of each syndrome were listed in a designated table. The Chinese Medicine practitioner collected data with the four classic diagnostic methods, completed the table, and diagnosed the syndrome of patient instantaneously.

### 2.3. Statistical Analyses

The data were entered into the Statistical Package for Social Sciences programme (SPSS 13.0), while the completed questionnaires were kept in a locked cabinet inside the clinic. Continuous variables were calculated using analysis of variance (ANOVA), and the chi-square test was used for analysing categorical data. All statistical tests were two-sided, and a *P* value of <0.05 was considered significant.

## 3. Results

A total of 228 advanced cancer patients were recruited. Three patients were excluded: two refused to participate and one could not complete questionnaire during interview. Thirty-nine participants, who could not write, only gave their verbal informed consent. For the 225 cases further analyzed, 127 patients (56.4%) and 7 patients (3.1%) were in deficient and excessive patterns, respectively, while 91 patients (40.4%) were in deficiency-excess complex. Deficiency-excess complex was a pathological state in which both deficiency and excess syndromes existed in the disease process [3]. The distributions of the five syndromes were: *Qi* deficiency (93.3%), *Qi* stagnation (40.0%), blood (*Yin*) deficiency (28.9%), *Yang* deficiency (22.2%), and excess heat (5.8%).

### 3.1. Demographic Data

Among the total of 225 patients, there were 119 males and 106 females. The three most common primary cancers were lung, colorectal, and hepatobiliary, accounting for 52.9% of the total. Patients in deficient pattern had the highest mean age at 75.82 years (SD = 11.21) and lowest PPS score at 55.69 years (SD = 16.18), while those in excessive pattern had the lowest mean age at 67.14 years (SD = 9.17) and highest PPS score at 71.67 (SD = 17.22), with *P* value < 0.05. However, there were no significant differences in gender and type of primary cancer. For the biochemical parameters, the level of blood haemoglobin, but not for urea, creatinine, alkaline phosphatase, alanine aminotransferase, calcium, and albumin, had significant differences among three groups. Patients in excessive pattern had the highest level of blood haemoglobin at 11.63 mg/dL (SD = 1.33), while those in deficient pattern had the lowest level at 10.20 mg/dL (SD = 2.02), with *P* value = 0.008 (Table 1).

### 3.2. Manifestations and Distributions for Patients in the Five Syndromes

The syndromes of excess heat, *Qi* stagnation, *Qi* deficiency, blood (*Yin*) deficiency and *Yang* deficiency were differentiated by the Chinese Medicine practitioner, and coexistence was allowed for patients with symptoms and signs complicated from more than one syndrome. The dominant manifestations of patients in each syndrome, with prevalence ≥50%, were listed in Table 2. Dry mouth, fatigue, and fine pulse were the common manifestations for patients with these five syndromes. Besides, the pattern of *Qi* deficiency was the fundamental syndrome among advanced cancer patients with constipation, with prevalence of 93.3%. For further analyzing its combination with other syndromes, one-third were in pure *Qi* deficiency, another one-third were coexistence with *Qi* stagnation or blood (*Yin*) deficiency, and the rest were in different combinations between the five syndromes (Table 3).

### 3.3. Manifestations for Patients in the Three Patterns

The prevalence of symptoms and signs for patients in deficient pattern, excessive pattern, and deficiency-excess complex was determined. The manifestations of pale/sallow complexion, fatigue, mind disquieted/susceptible to fright and anorexia among patients were significantly higher in deficient pattern than that in excess, with *P* value < 0.05. On the contrary, bitter taste, belching/nausea/vomiting, stuffiness and fullness of chest, abdominal distension/pain, water intention, insomnia, and vacuous pulse were more common in excessive pattern than that in deficiency, with *P* value < 0.05. For the description of constipation symptoms, patients in deficient pattern reported significantly higher prevalence of inadequate pushing force (42.5%), sense of incomplete defecation (15.7%), and difficulty in defecation (24.4%) when comparing with the group in excess (Table 4).

### 3.4. Patients' Severity of Constipation and Prescription of Opioid

The severity of constipation was significantly different among the three groups of patients with the most severe in the group of deficiency-excess complex (3.91 ± 1.57 points) and least in the deficient pattern (3.19 ± 1.85 points) (*P* = 0.012). About 50% patients in deficient pattern and deficiency-excess complex were in constipation, while patients in excessive pattern showed a discrete distribution of severity of constipation, for which 42.9% were in non-constipation and severe constipation, respectively. For the prescription of opioids, there were no significant difference on Syndrome distribution for whether patients were prescribed morphine, methadone, fentanyl, tramadol, dihydrocodeine, dextropropoxyphene or codeine, with *P* value > 0.05 (Table 5).

## 4. Discussion

From the results of this study, more than 90% of patients presented in deficient pattern, while 40% were in deficiency-excess complex. It illustrated that the healthy *Qi* (a collective designation for all normal functions of the human body and the abilities to maintain health [3]) of advanced cancer patients was greatly damaged, and many of them were complicated by excessive pattern. The excess condition could be caused by the accumulation of pathological factors, such as *Qi*, blood, phlegm, food, and dampness. Only a small number of patients were in pure excessive pattern. Therefore, the treatment principle of advanced cancer patients with constipation should reinforce the deficiency and eliminate the excess condition simultaneously. For the distribution of five common syndromes, two-thirds of patients present in *Qi* deficiency, or its combination with *Qi* stagnation and blood (*Yin*) deficiency, respectively. A designated TCM formula targeting on replenishing the deficiency of *Qi*, redirecting the flow of *Qi* stagnation and moistening the dryness caused by the blood (*Yin*) deficiency should be effective for the management of constipation in palliative care.

For analyzing the prevalence of symptoms and signs, there are many coincidences between different syndrome patterns. For example, dry mouth, fatigue, and fine pulse were the common manifestations of the five syndromes (Table 2). Even there were significant differences between deficient and excessive patterns, a large proportion of cases was actually in deficiency-excess complex (Table 4). It is not only because the specificity of symptom and sign for a particular syndrome pattern is relatively low, but also human body is an organic and complex whole, for which coexistence and transition of syndrome patterns are ordinary. Therefore, syndrome pattern should be differentiated comprehensively from a series of symptoms and signs. The dominant manifestations listed in Table 2 showed the norm of each symptom, which can be a reference for other TCM studies on advanced cancer.

In the past decades, the essence of syndrome patterns is determined with modern medical examination in terms of system biology [19]. For example, Chu et al. used serum proteomes to distinguish the essential hypertension patients with abundant phlegm-dampness from the healthy persons and the essential hypertension patients with non-phlegm-dampness [20]. In this study, patients in deficient pattern was significantly in higher mean age, and lower functional status (PPS) and level of blood haemoglobin when comparing with that in excessive pattern, while their Complex was at intermediate (*P* < 0.05). These objective assessment measures in conventional Western medicine may be as an auxiliary for the differentiation of syndrome patterns, although further investigations are necessary to develop certain guidelines. Furthermore, the impact of prescribed opioids on syndrome patterns cannot be concluded in this study. The influence of confounding by the opioid dose, duration of opioids intake, polypharmacy and polytreatment may be probably present.

Syndrome is the quintessence of TCM theory. However, consensus on its diagnosis is still limited; the diagnostic consistency among Chinese Medicine practitioners can be as low as 30% [21]. These not only make syndrome difficult to interpret and repeat on researches, but also impede the generalization of TCM to the world. We believe that standardizing the terminology, quantizing the description of syndrome, and validating TCM with evidence-based approaches are urgently needed for the development of TCM in future. Moreover, TCM has its vantage on analyzing diseases from a macroscopic point of view and human-oriented mind. Subjective measures, such as inadequate pushing force, sense of incomplete defecation and difficulty in defecation used in this study, are important for making diagnosis and prescription in TCM. On the contrary, conventional medicine is more dependent on objective measures and scientific assessment tools. Up till recently, patient's own perception of difficult defecation in the clinical assessment of constipation in palliative care and in treatment evaluation is emphasized [22]. We foresee that there are many opportunities for the incorporation of traditional medicine to convention medicine on diagnosis and treatment in both clinical practice and scientific researches.

Two aspects of this study should be reported as potential limitations in drawing broad conclusions. First, the whole study only involved 225 cases from two palliative care units, and there were 13 and seven patients in the groups of excess Heat and pure excessive pattern, respectively. When working with these small sample sizes, the results obtained from statistics may be underpowered to detect important effects or associations [23]. The distribution of syndrome patterns may not be able to generalize for all palliative cancer patients in Hong Kong. Second, variations in diagnosis do exist among CM practitioners [24]. Therefore, the diagnosis of syndrome should be made from more than one CM practitioner, and disagreements are resolved by discussions.

## 5. Conclusion

Advanced cancer patients were subject to be in deficient pattern, and many of them were complicated by excessive pattern. *Qi *deficiency and its combination of* Qi *stagnation and blood (*Yin*) deficiency were the most common syndromes for patients with constipation. Furthermore, age, functional status, and level of blood haemoglobin were factors related to the type of TCM syndrome. A TCM prescription with the functions on replenishing the deficiency, redirecting the flow of *Qi* stagnation and moistening the dryness caused by the blood (*Yin*) deficiency can be made for the treatment of advance cancer patients with constipation. Robust trials are urgently needed for further justifying its efficacy and safety in evidence-based approaches.

## Supplementary Material

This questionnaire was designed by Traditional Chinese Medicine professionals and Palliative Medicine specialists, comprising of three parts written in Chinese. The first part consisted of patient demographics, while the second part was about patients' perception of bowel function. The third part consisted of the traditional Chinese medicine (TCM) syndrome patterns, i.e. Excess Heat, Qi Stagnation, Qi Deficiency, Yang Deficiency and Yin Deficiency. Typical symptoms and signs of each syndrome were listed in a designated table. The Chinese Medicine practitioner collected data with the four classic diagnostic methods, completed the table, and diagnosed the syndrome of patient instantaneously.

## Figures and Tables

**Table 1:** Patient demographic data.

	Deficiency (*n* = 127)	Excess (*n* = 7)	Complex (*n* = 91)	*P* value
Gender ratio male: female	1 : 0.84	1 : 0.75	1 : 0.98	0.837
Age in years (mean ± SD)	75.82 ± 11.21	67.14 ± 9.17	72.57 ± 12.85	0.039
PPS (0–100) (mean ± SD)	55.69 ± 16.18	71.67 ± 17.22	60.23 ± 15.49	0.016

Primary cancer	Number of patients (% within group)			

Lung	38 (29.9%)	3 (42.9%)	24 (26.4%)	0.898
Colorectal	11 (8.7%)	1 (14.3%)	16 (17.6%)	
Hepatobiliary	16 (12.6%)	1 (14.3%)	9 (9.9%)	
Prostate	9 (7.1%)	0 (0%)	6 (6.6%)	
Stomach	11 (8.7%)	0 (0%)	3 (3.3%)	
Breast	7 (5.5%)	0 (0%)	5 (5.5%)	
Gynaecological	7 (5.5%)	0 (0%)	3 (3.3%)	
Pancreas	3 (2.4%)	0 (0%)	6 (6.6%)	
Urinary system	4 (3.1%)	1 (14.3%)	4 (4.4%)	
Nasopharyngeal	3 (2.4%)	0 (0%)	1 (1.1%)	
Thyroid	3 (2.4%)	0 (0%)	1 (1.1%)	
Haematological	3 (2.4%)	0 (0%)	1 (1.1%)	
Oesophagus	2 (1.6%)	0 (0%)	1 (1.1%)	
Brain	2 (1.6%)	0 (0%)	1 (1.1%)	
Head and neck	2 (1.6%)	0 (0%)	0 (0%)	
Others	3 (2.4%)	0 (0%)	4 (4.4%)	
Unknown/missing data	3 (2.4%)	1 (14.3%)	6 (6.6%)	

Biochemical parameters (mean ± SD)				

Haemoglobin mg/dL	10.20 ± 2.02	11.63 ± 1.33	11.00 ± 1.94	0.008
Urea mmol/L	5.90 ± 3.49	5.15 ± 3.54	7.01 ± 5.02	0.140
Creatinine *μ*mol/L	90.71 ± 47.19	70.50 ± 58.06	97.10 ± 62.50	0.425
Alkaline phosphatase IU/L	200.59 ± 256.56	187.67 ± 151.49	234.82 ± 275.38	0.711
Alanine aminotransferase U/L	36.08 ± 69.48	40.50 ± 15.98	88.64 ± 449.30	0.445
Serum calcium mmol/L	2.26 ± 0.27	2.15 ± 0.25	2.22 ± 0.17	0.243
Serum albumin mg/L	26.74 ± 6.27	23.00	27.44 ± 6.46	0.712

Deficiency: deficient pattern; excess: excessive pattern; complex: deficiency-excess complex.

PPS: palliative performance scale.

Biochemical parameters were determined for those patients with blood tests within three months.

**Table 2:** Dominant symptoms and signs of the five syndromes.

Excessive pattern:	
Excess heat: 13 patients (5.8%)	
Dry mouth 12/13 (92.3%), fatigue 10/13 (76.9%), phlegm production 7/13 (53.8%), slimy fur 8/13 (61.5%), white fur 8/13 (61.5%), fine pulse 7/13 (53.8%), and string-like pulse 9/13 (69.2%)	

*Qi* Stagnation: 90 patients (40.0%)	
Dry mouth 69/90 (76.7%), fatigue 81/90 (90.0%), mind disquieted/susceptible to fright 49/90 (54.4%), belching/nausea/vomiting	
61/90 (67.8%), abdominal distension/pain 58/90 (64.4%), anorexia 46/90 (51.1%), inadequate pushing force 56/90 (62.2%), pale red tongue 51/90 (56.7%), white fur 60/90 (66.7%), fine pulse 64/90 (71.1%), and string-like pulse 47/90 (52.2%)	

Deficient patterns:	
*Qi* deficiency: 210 patients (93.3%)	
Dry mouth 148/210 (70.5%), fatigue 200/210 (95.2%), mind disquieted/susceptible to fright 114/210 (54.3%), inadequate pushing force 110/210 (52.4%), pale red tongue 116/210 (55.2%), white fur 139/210 (66.2%), and fine pulse 147/210 (70.0%)	

Blood (*Yin*) deficiency: 65 patients (28.9%)	
Dry mouth 57/65 (87.7%), fatigue 62/65 (95.4%), mind disquieted/susceptible to fright 45/65 (69.2%), anorexia	
43/65 (66.2%), inadequate pushing force 38/65 (58.5%), red tongue 51/65 (78.5%), scanty fur/peeling fur/peeled fur 48/65(73.8%), and fine pulse 45/65 (69.2%)	

*Yang* deficiency: 50 patients (22.2%)	
Dry mouth 39/50 (78.0%), fatigue 48/50 (96%), mind disquieted/susceptible to fright 32/50 (64.0%), cold intolerance 41/50 (82.0%), pale red tongue 25/50 (50%), white fur 29/50 (58.0%), and fine pulse 33/50 (66.0%)	

Dominant symptoms and signs were defined as prevalent for more than or equal to 50% of each syndrome.

**Table 3:** The combination of deficiency of *Qi* with other syndromes.

Patients with *Qi* deficiency	Number of patients (%) 210/225 (93.3%)
*Qi* deficiency (pure)	70/210 (33.3%)
Coexistence with *Qi* deficiency	
*Qi* stagnation	39/210 (18.6%)
Blood (*Yin*) deficiency	31/210 (14.8%)
*Yang* deficiency	18/210 (8.6%)
Excess heat	5/210 (2.4%)
*Qi *stagnation and blood (*Yin) *deficiency	16/210 (7.6%)
*Qi *stagnation and *Yang* deficiency	14/210 (6.7%)
*Qi s*tagnation and excess heat	4/210 (1.9%)
Deficiency of blood (*Yin*) and* Yang *	6/210 (2.9%)
*Qi *stagnation and deficiency of blood (*Yin*) and *Yang *	6/210 (2.9%)
*Qi *stagnation, excess heat, and deficiency of blood (*Yin*) and *Yang *	1/210 (0.5%)

**Table 4:** Prevalence of symptoms and signs among three patterns.

	Deficiency (*n* = 127)	Excess (*n* = 7)	Complex (*n* = 91)	*P* value
Pale/sallow complexion	59 (46.5%)	0 (0%)	28 (30.8%)	0.007
Bitter taste	28 (22.0%)	2 (28.6%)	37 (40.7%)	0.012
Fatigue	122 (96.1%)	4 (57.1%)	82 (90.1%)	<0.001
Mind disquieted/susceptible to fright	66 (52.0%)	0 (0%)	50 (54.9%)	0.019
Belching/nausea/vomiting	18 (14.2%)	4 (57.1%)	57 (62.6%)	<0.001
Stuffiness and fullness of chest	5 (3.9%)	1 (14.3%)	40 (44.0%)	<0.001
Abdominal distension/pain	9 (7.1%)	1 (14.3%)	57 (62.6%)	<0.001
Water retention (with pleural/abdominal fluid)	0 (0%)	1 (14.3%)	13 (14.3%)	<0.001
Anorexia	47 (37.0%)	1 (14.3%)	46 (50.5%)	0.044
Insomnia	36 (28.3%)	4 (57.1%)	40 (44.0%)	0.029
Vacuous pulse	9 (7.1%)	3 (42.9%)	5 (5.5%)	0.001

Description of constipation symptoms				

Inadequate pushing force during defecation	54 (42.5%)	1 (14.3%)	58 (63.7%)	0.001
Incomplete defecation	20 (15.7%)	0 (0%)	27 (29.7%)	0.017
Difficult defecation	31 (24.4%)	1 (14.3%)	37 (40.7%)	0.024

Deficiency: deficient pattern; excess: excessive pattern; complex: deficiency-excess complex.

Only the symptoms and signs with significant differences among three syndrome patterns were listed in the table.

**Table 5:** The relationship between severity of constipation and opioids intake with the pattern distribution.

	Deficiency (*n* = 127)	Excess (*n* = 7)	Complex (*n* = 91)	*P* value
Severity (mean ± SD)	3.19 ± 1.85	3.29 ± 2.21	3.91 ± 1.57	0.012
No constipation	32 (25.2%)	3 (42.9%)	9 (9.9%)	0.013
Constipation	63 (49.6%)	1 (14.35%)	50 (54.9%)	
Severe constipation	32 (25.2%)	3 (42.9%)	32 (35.2%)	
With opioids	73 (57.5%)	4 (57.1%)	60 (65.9%)	0.442
With strong opioids	29 (22.8%)	2 (28.6%)	26 (28.6%)	0.618
With weak opioids	49 (38.6%)	2 (28.6%)	36 (39.6%)	0.847

Deficiency: deficient pattern; excess: excessive pattern; complex: deficiency-excess complex.

Severity of constipation was evaluated with an 8-point ordinal rating scale, where 0-1 indicated no constipation, 2–4 indicated constipation, and 5–7 indicated severe constipation.

Strong opioids included morphine, methadone, and fentanyl, while weak opioids included tramadol, dihydrocodeine, dextropropoxyphene, and codeine.
