# Supplementary material for: The Quintessence of Traditional Chinese Medicine: Syndrome and Its Distribution among Advanced Cancer Patients with Constipation
Source: Evid Based Complement Alternat Med. 2012 Jun 24;2012:739642. doi: 10.1155/2012/739642 (PMC3388626; doi:10.1155/2012/739642)
Supplement: Supplementary file 1 — This questionnaire was designed by Traditional Chinese Medicine professionals and Palliative Medicine specialists, comprising of three parts written in Chinese. The first part consisted of patient demographics, while the second part was about patients' perception of bowel function. The third part consisted of the traditional Chinese medicine (TCM) syndrome patterns, i.e. Excess Heat, Qi Stagnation, Qi Deficiency, Yang Deficiency and Yin Deficiency. Typical symptoms and signs of each syndrome were listed in a designated table. The Chinese Medicine practitioner collected data with the four classic diagnostic methods, completed the table, and diagnosed the syndrome of patient instantaneously. [file 739642.f1.doc]

**Site地點: ____CMC / OLMH____**

**Subject code編號: ________________________**

**Date日期: ________________________**

**Age年齡: ________________________**

**Gender性別 : ________________________**

**Screening Table篩選表**

| **Inclusion criteria納入標準** | **Yes是** | **No否** |
| --- | --- | --- |
| 1) Aged 18 years or above 年滿18歲或以上 |  |  |
| 2) Subject with advanced cancer enrolled in palliative care 末期癌症患者 |  |  |
|  |  |  |
| **Exclusion criteria排除標準** |  |  |
| 1. Unable to communicate (e.g. cognitive impairment) 溝涌障礙 |  |  |
| 1. Constipation is not confirmed未能確定便秘問題 |  |  |
| 1. Recognized as at end-of-life (EOL) and laxatives / enemas are discontinued EOL並停止使用瀉藥/灌腸劑患者 |  |  |
| 1. Suspected to have gastrointestinal obstruction懷疑消化道阻塞患者 |  |  |
|  |  |  |
| **Remarks備註** |  |  |
| Interested to take part of the clinical study 有否有興趣參與臨床研究 |  |  |

**Result結果: Included納入 / Excluded排除, Reason原因:___________________________**

**Investigator研究人員: _______________ Sign簽署: _______________ Date日期:__________**

**Part I Medical History 病歷資料**

Recruitment Date:

| **Type of cancer**  **癌症類型** |  | |
| --- | --- | --- |
| **Date of diagnosis**  **確診日期** |  | |
| **Opioid intake 服用止痛藥**  **-Start from 開始:** | **Yes 是 / No 否 Daily dose:**  **(yy/mm/dd) (年年/月月/日日)** | |
| **Current medications**  **服用藥物** |  | |
| **Functional status 功能狀態**  **(PPS score評分)** | **/ 0~100%** | |
| **Last laboratory results**  **最近相關化驗結果**  **Data best within:** | **Hb:**  **Urea: Creat:**  **Alb:**  **Ca:** | **AST: ALT:**  **ALP:**  **Other:** |

**Part II Bowel Function 腸道功能**

| **Bowel frequency**  **排便次數** | **/ week星期** |
| --- | --- |
| **Stool type大便類型**  **(Bristol stool scale)** | **/ 1~7** |
| **Rectal measure required手法協助排便**  **-Frequency次數** | **Yes是 / No否**  **/ week星期** |
| **Laxatives/enemas required使用瀉藥/灌腸劑**  **-Frequency次數** | **Yes / No**  **/ week星期** |
| **How would you rate your severity of constipation?**  **自覺便秘問題嚴重程度**  **None無 0 1 2 3 4 5 6 7 Most severe最嚴重** | |

**Part III TCM syndromes中醫證候分析**

| 症狀 | | 證型 | | | | | | |
| --- | --- | --- | --- | --- | --- | --- | --- | --- |
|  |  | 腸道實熱 | 腸道氣滯 | | 脾虛氣弱 | 脾腎陽虛 | 陰虛腸燥 | 其他 |
| 大便性質 | 質 | □ 乾硬 □ 硬 □ 稍乾 □ 正常 □ 軟 □ 爛 □ 水樣 | | | | | |  |
|  | 形 | □ 粒狀 □ 粒狀結成短條 □ 條狀表面凹凸不平 □ 正常條狀  □ 偏散 □ 稀爛 □ 水樣 | | | | | |  |
|  | 味 | □ 臭穢 □ 正常 □ 腥穢 | | | | | |  |
| 主證 | | □ 臨厠努掙 | | □ 排便不盡  □ 艱澀不暢 | □ 臨厠努掙乏力 | □ 臨厠努掙乏力 | □ 臨厠努掙乏力 |  |
|  |  | □ 腹中脹滿  □ 腹中痛 / 按之作痛 | | | □ 神疲  □ 氣怯 | □ 身寒肢冷  □ 小腹冷痛 | □ 口乾少津 |  |
|  |  | □ 口乾  □ 口苦  □ 口臭 | | □ 噯氣頻作  □ 胸脇痞滿 | □ 舌淡  □ 舌胖齒印 | □ 眩暈  □ 心悸 | □ 舌紅  □ 少苔 |  |
| 兼夾證 | | □ 面紅 | | □ 與情志相關 | □ 面色無華 | □ 面色(白光)白 | □ 顴紅 |  |
|  |  | □ 小便短赤 | | □ 舌淡紅  □ 苔白 | □ 汗出  □ 氣短 | □ 小便清長 | □ 神疲  □ 納呆 |  |
|  |  | □ 舌紅  □ 苔黃 | | □ 脈弦  □ 脈細 | □ 脈細  □ 脈弱 | □ 舌淡  □ 苔白潤 | □ 脈細  □ 脈數 |  |
| 其他 | | □ 脈滑  □ 脈實 | |  |  | □ 脈沉  □ 脈遲 |  |  |
| **分析**  **結果** | |  | |  |  |  |  |  |
